# Intraoperative hypotension after remimazolam or propofol induction with sevoflurane maintenance in angiotensin II receptor blockers-treated patients: a randomized controlled trial

**DOI:** 10.1038/s41598-025-23469-y

**Published:** 2025-11-13

**Authors:** Hye Jin Kim, Namo Kim, Jiho Kim, Jin Ha Park, Hye Jung Shin, Jinho Yang, So Yeon Kim

**Affiliations:** 1 Department of Anesthesiology and Pain Medicine, Anesthesia and Pain Research Institute, Yonsei University College of Medicine, 50-1 Yonsei-ro, Seodaemun-gu, Seoul, 03722 Republic of Korea; 2 Department of Biomedical Systems Informatics, Biostatistics Collaboration Unit, Yonsei University College of Medicine, Seoul, Republic of Korea

**Keywords:** Angiotensin receptor antagonists, Propofol, Remimazolam, Hypotension, Anesthesia, Anesthetics, intravenous, Diseases, Medical research

## Abstract

Robot-assisted laparoscopic prostatectomy (RALP) is associated with hemodynamic shifts; patients on angiotensin receptor blockers (ARBs) are especially susceptible to intraoperative hypotension. We investigated whether induction with remimazolam reduces the intraoperative hypotension incidence and severity compared with propofol in this population. Herein, 112 hypertensive patients undergoing RALP who continued ARB therapy received remimazolam (0.2 mg/kg) or propofol (1–1.5 mg/kg) for anesthetic induction. The primary endpoint was hypotension occurrence (mean arterial pressure [MAP] < 65 mmHg sustained for ≥ 1 min), assessed during the entire anesthesia and 15 min post-induction. Secondary endpoints included MAP < 55 mmHg sustained for ≥ 1 min, time-weighted average (TWA)-MAP < 65 mmHg or < 55 mmHg, duration of MAP < 65 mmHg, and required norepinephrine dose. The hypotension incidence did not differ significantly between groups during the entire anesthesia (87.5% vs. 89.3%, *P* > 0.999). During the entire anesthesia, no significant between-group differences were observed for MAP < 55 mmHg for ≥ 1 min, TWA-MAP < 65 mmHg or < 55 mmHg, duration of MAP < 65 mmHg, and required norepinephrine dose. Similarly, no significant between-group differences were observed during the 15 min after induction. Induction with remimazolam did not reduce intraoperative hypotension risk compared with low-dose propofol in patients undergoing RALP who continued ARB therapy.

**Clinical trial registration number**: ClinicalTrials.gov (NCT06093971, 23/10/2023).

## Introduction

The use of angiotensin II receptor blockers (ARBs) in the management of hypertension has steadily increased due to their efficacy and favorable safety profile^1^. However, perioperative management of ARBs in non-cardiac surgery—particularly whether to continue or withhold them on the day of surgery—remains a subject of ongoing debate^2–8^. Many patients continue ARB therapy on the day of surgery, which has been associated with a higher risk of intraoperative hypotension compared to those who discontinue therapy 24 h preoperatively^9^.

Robot-assisted laparoscopic prostatectomy (RALP) is known to cause substantial hemodynamic changes as a result of pneumoperitoneum and the steep Trendelenburg position^10,11^. Patients continuing ARB therapy may be especially susceptible to these effects, making the identification of anesthetic strategies to reduce intraoperative hypotension an important clinical priority.

Remimazolam, a benzodiazepine-based anesthetic agent, has shown promise as an alternative to propofol^12^. Its rapid onset and offset, combined with favorable hemodynamic stability, suggest potential advantages in patients at elevated risk of intraoperative hypotension^13^. Although postinduction hypotension alone may not be independently associated with complications, its clinical relevance increases when it persists long enough to contribute to the overall intraoperative hypotension burden^14^. Therefore, we evaluated two periods of hypotension: hypotension occurring during the entire anesthesia and within 15 min of anesthetic induction.

## Methods

### Ethics

This trial adhered to the Declaration of Helsinki and was approved by the Institutional Review Board of Severance Hospital, Yonsei University Health System (IRB no. 4-2023-0803, August 2023). It was registered at ClinicalTrials.gov (NCT06093971, 23/10/2023) prior to patient enrolment. Written informed consent was obtained from all participants. The study follows the Consolidated Standards of Reporting Trials guidelines.

### Study design and patients

This single-center, parallel-group, randomized controlled trial enrolled patients undergoing RALP at Severance Hospital between November 2023 and October 2024. Inclusion criteria were hypertensive patients aged ≥ 19 years who had been receiving ARBs for at least 3 months. Exclusion criteria included emergency surgery, inability to read or understand the consent form (e.g., illiteracy or language barrier), cognitive impairment, atrial fibrillation, moderate to severe valvular disease, and a left ventricular ejection fraction ≤ 35%. Patients who did not receive ARBs on the day of surgery were also excluded.

Participants were withdrawn from the study if they: (1) withdrew consent; (2) underwent a change in planned surgical procedure; (3) experienced blood pressure data collection failure during surgery, resulting in a data gap of ≥ 5 min; or (4) could not undergo arterial line insertion prior to anesthesia induction.

### Randomized allocation and blinding

N. Kim generated the random allocation sequence using Microsoft Excel 2016^®^ (Microsoft Corp., Redmond, WA, USA), applying a fixed block size of four with a 1:1 allocation ratio. Patients were assigned to either the propofol or remimazolam group. Both agents were prepared by a researcher not involved in anesthesia care, data collection, or analysis; remimazolam was diluted to a concentration of 1 mg/mL. The initial and subsequent doses were drawn into separate syringes and placed in an opaque plastic box to ensure concealment. To maintain blinding, the infusion line from the injection site to the patient’s intravenous cannula was covered with a surgical drape. All drugs were administered behind an opaque mobile radiation-shielding screen (approximately 1.8 m in height and 0.9 m in width). This ensured that patients, anesthesiologists, data-collecting physicians, and surgeons remained blinded to group allocation.

### Anesthesia protocol

All patients underwent surgery in the morning and had been kept nil per os since midnight. No routine fluid loading was administered before or during induction. On arrival in the operating room, patients were monitored using standard devices, including a three-lead electrocardiogram, non-invasive blood pressure monitor, pulse oximeter, and SedLine^®^ monitor (Masimo Corp., Irvine, CA, USA). Ultrasound-guided radial artery cannulation was performed with a 20-gauge catheter following local infiltration with 1% lidocaine, and hemodynamic monitoring was initiated using the Acumen IQ sensor with the HemoSphere monitoring platform (Edwards Lifesciences, Irvine, CA, USA). After arterial cannulation, glycopyrrolate 0.1 mg was administered intravenously, and patients received 100% oxygen via face mask for at least 3 min.

Anesthesia induction began with remifentanil via target-controlled infusion (TCI). Once the effect-site concentration (Ce) reached 3.0 ng/mL, lidocaine 20 mg was administered intravenously. The propofol group received propofol (Fresofol^®^ 1% MCT, Fresenius Kabi Korea Ltd., Seoul, Korea) at 1.0 mg/kg, while the remimazolam group received remimazolam (Byfavo^®^; HanaPharm Co., Ltd., Hwaseong, Korea) at 0.2 mg/kg, administered over 30 s to 1 min. Additional doses of propofol (0.5 mg/kg) and remimazolam (0.1 mg/kg) were given if required. Following loss of consciousness, 1.2 mg/kg of rocuronium (Rocumeron^®^; Ilsung Pharmaceuticals Co., Ltd., Seoul, Korea) was administered, followed by mask ventilation with 2% sevoflurane.

Tracheal intubation was performed after confirming the absence of response to 50-Hz train-of-four stimulation of the ulnar nerve at the adductor pollicis muscle using a peripheral nerve stimulator (TwitchView^®^; Blink Device Co., Seattle, WA, USA). Mechanical ventilation settings included a tidal volume of 7–8 mL/kg ideal body weight and a positive end-expiratory pressure of 5 cmH_2_O. The respiratory rate was adjusted to maintain end-tidal carbon dioxide (CO_2_) between 40 and 50 mmHg. Anesthesia was maintained with sevoflurane (0.8–1.0 MAC) and remifentanil (TCI, Ce 1.0–2.0 ng/mL) to maintain a Patient State Index between 25 and 50. Norepinephrine was infused if mean arterial pressure (MAP) dropped below 65 mmHg, and ephedrine 4 mg was administered if heart rate (HR) fell below 50 bpm. All RALPs were performed by a single surgeon. Pneumoperitoneum was induced with CO_2_ insufflation, and patients were placed in a 30° Trendelenburg position with an intra-abdominal pressure of 12 mmHg.

### Data collection

Baseline demographic and clinical data were collected, including age, sex, height, weight, American Society of Anesthesiologists (ASA) physical status, medical history, and medications. Hemodynamic data were extracted from the HemoSphere monitor at 20-s intervals. Additional intraoperative data included the duration of anesthesia, fluid intake volume, urine output, blood loss, and the administered doses of remifentanil, ephedrine, and norepinephrine. Postoperative complications within 30 days were prospectively assessed, including pneumonia, acute kidney injury (AKI)^15^, myocardial infarction, stroke, unexpected return to the operating room, and mortality.

### Study endpoints

The primary endpoint was the occurrence of hypotension, defined as an MAP < 65 mmHg sustained for ≥ 1 min. Hypotension was assessed across two distinct periods: (1) the entire duration of anesthesia and (2) the 15 min following initiation of induction with propofol or remimazolam (post-induction).

Secondary endpoints included the occurrence of MAP < 55 mmHg sustained for at least 1 min, duration of MAP < 65 mmHg, and total dose of norepinephrine administered. Additionally, the severity of hypotension, measured as the time-weighted average (TWA)-MAP < 65 or 55 mmHg which was calculated as the area under the MAP value of 65 or 55 divided by the total measurement time (min), was assessed as a secondary endpoint.

### Sample size calculation

A subgroup analysis from our prior study using propofol for anesthetic induction of RALP found an incidence of hypotension (MAP < 65 mmHg sustained for 1 min) of 83.5% in patients receiving preoperative ARBs^16^. Assuming that remimazolam induction could reduce this incidence by 30% (relative reduction)^12,17^, a sample of 50 patients per group was required to detect a difference of 0.25 between the null hypothesis (0.835) and alternative hypothesis (0.585) with α = 0.05 and power = 80%. Allowing for a 10% dropout rate, a total of 112 participants were enrolled (*n* = 56 per group).

### Statistical analysis

There were no missing data. Normality of distribution for continuous variables was assessed using the Shapiro–Wilk test. Depending on distribution, comparisons were made using the independent two-sample t-test or Mann–Whitney U test. Normally distributed data are presented as mean (standard deviation [SD]), whereas non-normally distributed data are presented as median (interquartile range [IQR]). Categorical variables were compared using the chi-square test or Fisher’s exact test and reported as numbers (percentage).

Additionally, MAP, HR, stroke volume (SV), and cardiac output (CO) were compared between groups at 1-minute intervals during the 15-min post-induction. Independent two-sample t-test were performed at 16 time points for each interval (i.e., once per minute). The difference between MAP before anesthesia induction and the lowest MAP within 15 min after induction was evaluated using an independent two-sample t-test. To account for multiple comparisons, Bonferroni correction was applied, and adjusted p-values were calculated by multiplying the raw p-values by 16. All analyses were conducted using SPSS version 26 (IBM Corp., Armonk, NY, USA) and R version 4.3.2 (The R Foundation for Statistical Computing, Vienna, Austria). Statistical significance was defined as *P* < 0.05.

## Results

**Fig. 1:**
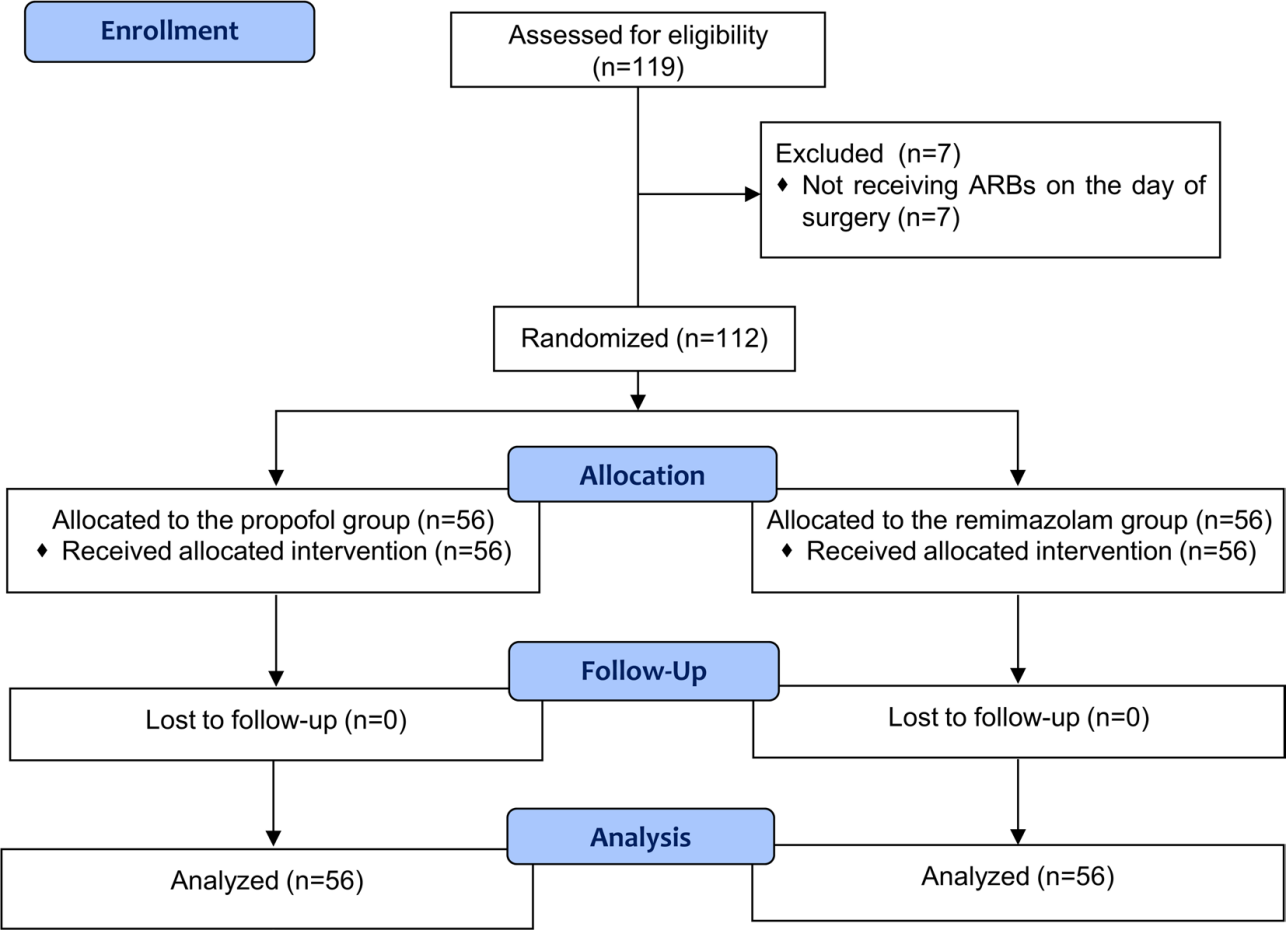
CONSORT diagram of patient recruitment.

Of the 119 patients screened for eligibility, 112 were enrolled and completed the study, with 56 patients in each of the propofol and remimazolam groups (Fig. 1). One patient in the propofol group required an additional induction dose of 0.5 mg/kg of propofol. There were no significant differences observed in the classification of ARB medications used (Supplementary Table S1). Demographic characteristics, preoperative medical histories, and other medication use were comparable between groups. Moreover, the duration of anesthesia, total fluid intake, blood loss, and total remifentanil dose administered were similarly comparable (Table 1).

**Table 1:** Baseline characteristics and intraoperative data. Values are presented as mean (standard deviation), median [interquartile range] or the numbers of patient (percentage). ASA, American society of Anesthesiologists; COPD, chronic obstructive lung disease.

Variables	Propofol (*n* = 56)	Remimazolam (*n* = 56)	*P*-value
Demographic factors			
Age (years)	68.3 (7.0)	66.9 (5.9)	0.250
Height (cm)	167.3 (4.6)	167.5 (5.7)	0.814
Weight (kg)	72.7 (9.1)	72.2 (7.7)	0.717
ASA physical status			0.440
II	49 (87.5)	45 (80.4)	
III	7 (12.5)	11 (19.6)	
Medical history			
Diabetes mellitus	21 (37.5)	19 (33.9)	0.844
COPD	5 (8.9)	5 (8.9)	> 0.999
Coronary artery disease	2 (3.6)	2 (3.6)	> 0.999
Chronic kidney disease	4 (7.1)	1 (1.8)	0.364
Old cerebrovascular accident	2 (3.6)	2 (3.6)	> 0.999
Preoperative medication			
Beta-blocker	4 (7.1)	2 (3.6)	0.679
Calcium channel blocker	33 (58.9)	36 (64.3)	0.698
Diuretics	6 (10.7)	10 (17.9)	0.418
Intraoperative data			
Anesthesia duration (min)	140 [125–155]	140 [124–161]	0.933
Fluid intake (mL)	1400 [1188–1613]	1450 [1288–1663]	0.179
Urine output (mL)	250 [200–400]	250 [200–400]	0.662
Blood loss (mL)	175 [100–400]	200 [100–400]	0.244
Remifentanil dose (µg)	382 [321–452]	384 [331–463]	0.845

The MAP [mean (SD)] measured after arterial cannulation and before induction was similar between the propofol and remimazolam groups: 103 (12) mmHg and 102 (11) mmHg, respectively (*P* = 0.713). Hemodynamic data during the entire anesthesia period are summarized in Table 2. The incidence of hypotension did not differ significantly between the propofol and remimazolam groups (49 [87.5%] vs. 50 [89.3%], *P* > 0.999), nor did the severity of hypotension, as measured by TWA-MAP < 65 mmHg (0.40 [0.07–0.87] mmHg vs. 0.31 [0.12–0.77] mmHg, *P* = 0.764). Other parameters, including the occurrence of MAP < 55 mmHg sustained for ≥ 1 min, TWA-MAP < 55 mmHg, duration of MAP < 65 mmHg, number of patients receiving ephedrine, and total norepinephrine dose administered per patient, were also comparable between groups. In addition, no significant between-group differences were found in any of these variables during the 15-min post-induction (Table 3).

**Table 2:** Hemodynamic data during whole anesthesia period. Values are presented as median [interquartile range] or the number of patients (percentage). MAP, mean arterial pressure; TWA, time-weighted average.

Variables	Propofol (*n* = 56)	Remimazolam (*n* = 56)	*P*-value
Patients with ≥ 1 consecutive min of MAP < 65 mmHg	49 (87.5)	50 (89.3)	> 0.999
Patients with ≥ 1 consecutive min of MAP < 55 mmHg	20 (35.7)	13 (23.2)	0.214
TWA-MAP < 65 mmHg (mmHg)	0.40 [0.07–0.87]	0.31 [0.12–0.77]	0.764
TWA-MAP < 55 mmHg (mmHg)	0.00 [0.00–0.07]	0.00 [0.00–0.02]	0.151
Duration of MAP < 65 mmHg (min)	11.83 [3.58–21.75]	10.83 [5.25–20.42]	0.963
Patients with ephedrine use	4 (7.1)	5 (8.9)	> 0.999
Ephedrine dose (mg)	0 [0–0]	0 [0–0]	0.697
Norepinephrine dose (µg)	193 [67–372]	164 [92–341]	0.871

**Table 3:** Hemodynamic data during the 15 min after the start of Propofol or remimazolam administration. Values are presented as median [interquartile range] or the number of patients (percentage). MAP, mean arterial pressure; TWA, time-weighted average.

Variables	Propofol (*n* = 56)	Remimazolam (*n* = 56)	*P*-value
Patients with ≥ 1 consecutive min of MAP < 65 mmHg	29 (51.8)	28 (50.0)	> 0.999
Patients with ≥ 1 consecutive min of MAP < 55 mmHg	12 (21.4)	8 (14.3)	0.459
TWA-MAP < 65 mmHg (mmHg)	0.33 [0.00–2.22]	0.16 [0.00–1.16]	0.517
Duration of MAP < 65 mmHg (min)	1.17 [0.00–6.33]	1.00 [0.00–3.75]	0.626

**Fig. 2:**
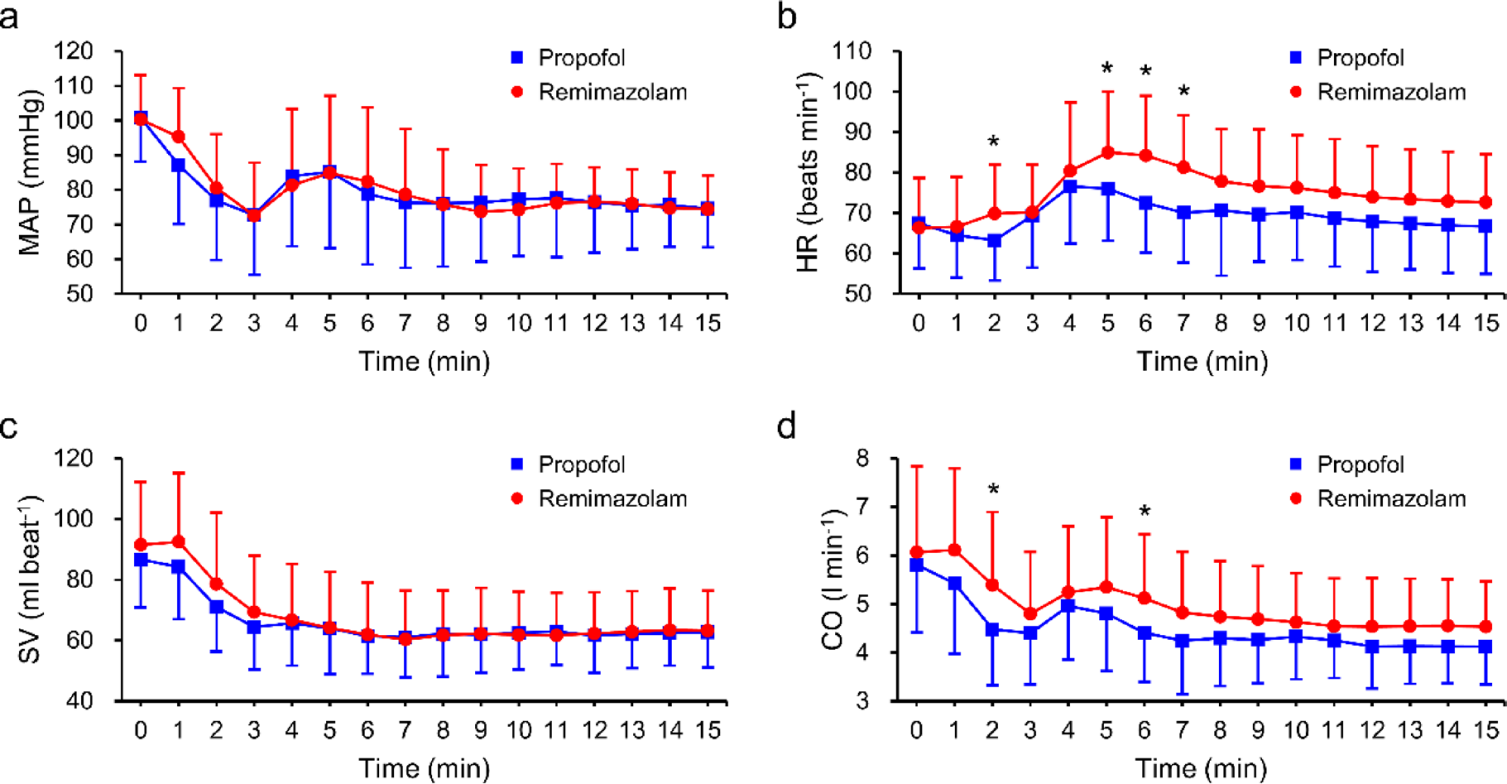
Changes in (**a**) mean arterial pressure (MAP), (**b**) heart rate (HR), (**c**) stroke volume (SV), and (**d**) cardiac output (CO) during the 15-min of post-induction period. Each panel presents mean and standard deviation (SD) plots for propofol (blue squares) and remimazolam (red circles), measured every minute from the start of induction to 15 min post-induction. Time = 0 represents the moment of induction agent administration. Between-group comparisons at each time point were performed using the independent two-sample t-test. Bonferroni correction was applied for multiple comparisons (adjusted P *=* raw P x 16). Asterisks (*) denote time points with an adjusted P-value < 0.05.

During the 15-min post-induction period, MAP and SV did not differ significantly between groups; however, HR and CO were slightly higher in the remimazolam group at the 2-min and approximately 6-min time points (Fig. 2). Moreover, the mean MAP decrease from the value before anesthesia induction to the lowest value within 15 min after induction was 39.2 ± 11.7 mmHg in the propofol group and 37.9 ± 10.7 mmHg in the remimazolam group, with no statistically significant difference between the groups (*P* = 0.527) (Supplementary Figure S1).

Postoperative complications occurred in only one patient in the propofol group, who developed AKI and required reoperation on postoperative day 4 for bowel perforation; this patient had intraoperative hypotension. Because morbidity occurred in only one patient, no statistical comparison was performed given the extremely low event rate.

## Discussion

In this parallel-group, randomized controlled trial, we found that bolus induction with remimazolam, compared with propofol, did not reduce intraoperative hypotension in patients undergoing RALP who continued ARB therapy on the day of surgery. This finding was consistent during both the post-induction and overall anesthesia periods. Furthermore, remimazolam did not reduce the frequency or total dose of vasopressors administered.

Preexisting hypertension and ARB use are both recognized risk factors for intraoperative hypotension, contributing to ongoing debate regarding the perioperative management of ARBs^18,19^. Withholding ARBs may reduce the risk of hypotension, though its clinical benefits remains uncertain^8^. A recent multicenter randomized controlled trial (RCT) found no increase in postoperative complications when ARBs were continued^5^. Another multicenter RCT reported that discontinuation failed to reduce myocardial injury and may increase the risk of significant perioperative hypertension^20^. Amid conflicting findings, the most recent AHA guidelines suggest that withholding ARBs for 24 h before elevated-risk non-cardiac surgery may be reasonable to reduce intraoperative hypotension^2^. However, these guidelines do not provide definitive recommendations for specific surgeries or patient populations^5^, and many patients still undergo surgery while continuing ARB therapy.

For these patients, clinicians must remain vigilant about managing intraoperative hypotension. RALP carries a particularly high risk of hemodynamic instability due to factors such as the advanced age of the surgical population and the hemodynamic fluctuations caused by steep Trendelenburg positioning and rapid CO₂ insufflation and desufflation. In our study, the mean patient age was approximately 65–70 years, and nearly 88% experienced hypotension (defined as ≥ 1 consecutive min of MAP < 65 mmHg) at some point during the anesthesia period. The median duration of MAP < 65 mmHg was 10 min, with an upper quartile of 20 min. Severe hypotension (≥ 1 consecutive min of MAP < 55 mmHg) occurred in 29% of patients. MAP values below 65 mmHg have been identified as a critical threshold for organ injury^21^, and sustained exposure to MAP < 65 mmHg for ≥ 10 min—or any exposure to MAP < 55 mmHg—is significantly associated with increased risk of end-organ injury following non-cardiac surgery^22^. Therefore, anesthetic strategies that promote hemodynamic stability may be essential in mitigating postoperative complications in vulnerable populations.

Remimazolam is an ultra-short-acting benzodiazepine that was first approved for procedural sedation during endoscopy and subsequently approved for use in general anesthesia^13^. It has been shown to reduce the incidence of hypotension during endoscopic sedation^23,24^. In addition, several studies have reported a lower risk of hypotension with remimazolam compared with propofol in both cardiac^25,26^ and non-cardiac surgery^12,17,27^. However, not all findings are consistent. Other studies have found no significant difference in the incidence of hypotension between remimazolam and propofol during anesthetic induction^28,29^.

In one study involving patients on angiotensin-converting enzyme inhibitors (ACEIs) or ARBs, remimazolam was associated with significantly less post-induction hypotension than propofol (62.5% vs. 82.9%, *P* = 0.04)^17^. In contrast, our study found no difference between groups in either the incidence or severity of hypotension during any intraoperative phase. A likely explanation for this discrepancy is the lower propofol dose used in our trial (1 mg/kg) compared with that study (2 mg/kg). Although the patients in the prior study were somewhat younger (mean age ≈ 60 years), a 2 mg/kg dose may exceed the appropriate range for older adults and contribute to hypotension. In our study, more than two-thirds of patients were aged ≥ 65 years and approximately 19% were aged ≥ 75 years, indicating that the cohort largely comprised an older surgical population. FDA guidance recommends 20 mg every 10 s (up to 1–1.5 mg/kg total) for induction in older patients or those classified as ASA physical status III or IV^30^. Because propofol requirements decline with age, a dose of 1 mg/kg is considered appropriate for patients aged ≥ 65 years^31^. Moreover, one study in patients receiving ACEIs found that each 0.3 mg/kg increase in propofol dose was associated with a 31% increase in hypotensive or bradycardic episodes requiring intervention^32^. These findings support the idea that careful titration of propofol in older patients may yield hemodynamic outcomes comparable to those seen with remimazolam. However, in our study, the age range was not restricted to a narrowly defined elderly subgroup (e.g., ≥ 75–80 years), and further studies focusing on more age-restricted cohorts are warranted to clarify the influence of age on hemodynamic responses.

Our study is the first to investigate serial changes in SV and CO following remimazolam administration using arterial pulse waveform analysis. Arterial cannulation was performed prior to the administration of propofol or remimazolam, allowing direct comparison of hemodynamic indices between groups. MAP values were comparable during the 15-min post-induction period; however, CO was marginally higher in the remimazolam group at approximately 2 and 6 min post-induction, coinciding with a transient increase in HR. As SV remained stable, the rise in CO appears to have been driven primarily by the increase in HR. Previous studies have also reported that remimazolam tends to elicit a higher HR than propofol during the induction period in older adults^29^ and in patients undergoing neurosurgical procedures^33^. However, the clinical significance of these small differences in HR and CO appears limited.

Our findings have several implications for anesthetic management in patients who continue ARB therapy. First, the comparable hemodynamic profiles observed between bolus low-dose propofol and remimazolam suggest that, with careful titration, propofol induction may remain a viable option in this high-risk population, challenging the common perception that propofol may be avoided in ARB-treated patients. Second, although remimazolam has demonstrated superior hemodynamic stability in other clinical settings^23–27^, its benefit may be limited in patients with continued ARB therapy, particularly when administered as a bolus. Finally, the high incidence of intraoperative hypotension in both groups underscores the need for proactive hemodynamic strategies—such as pre-emptive vasopressor support, and careful fluid management—regardless of the induction agent used.

This study has several limitations. First, the question of dose equivalence between propofol and remimazolam remains. Given the high-risk profile of our study population, we selected a low propofol dose (1 mg/kg) in accordance with FDA recommendations^30^. Only one participant required 1.5 mg/kg—the upper limit recommended for older patients. Second, remimazolam was administered as a bolus, whereas continuous infusion is more common for induction at rates of 6 or 12 mg/kg/h^13^. We based our protocol on the study by Chae et al.^34^, which demonstrated the safety and efficacy of bolus administration. Accordingly, we used a dose of 0.2 mg/kg—comparable to the ED95 for loss of consciousness (0.19 mg/kg) in 60-year-old patients—and extended the injection time to 30–60 s, exceeding the standard 20-s administration window. This corresponds to an infusion rate of 12–24 mg/kg/h, which is modestly above the traditional induction infusion rate of 6 or 12 mg/kg/h. Although continuous infusion is an ideal option, bolus administration remains common in clinical practice, and our protocol was designed to balance patient safety with real-world applicability. Third, concomitant antihypertensive agents other than ARBs may have influenced blood pressure; however, their comparable baseline distribution between groups reduces the likelihood of meaningful confounding. Finally, the study may have been underpowered. Our sample size was based on an anticipated 30% relative reduction in hypotension with remimazolam, and this assumption may have been overly optimistic. Larger trials are needed to validate our findings and assess potential subgroup effects.

In conclusion, bolus administration of remimazolam (0.2 mg/kg) did not reduce the incidence of hypotension compared with low-dose propofol (1–1.5 mg/kg) in patients undergoing RALP while continuing ARB therapy. Although remimazolam has shown superior hemodynamic stability in other settings, these findings suggest that with careful titration, low-dose propofol may achieve comparable hemodynamic outcomes in patients continuing ARB therapy. Nonetheless, the high incidence of intraoperative hypotension in both groups emphasizes the ongoing hemodynamic challenges in this patient population.

## Supplementary Information

Below is the link to the electronic supplementary material.

Supplementary Material 1

## Data Availability

The datasets generated during the current study are available from the corresponding author on reasonable request.
