# Supplementary material for: Intraoperative hypotension after remimazolam or propofol induction with sevoflurane maintenance in angiotensin II receptor blockers-treated patients: a randomized controlled trial
Source: Sci Rep. 2025 Nov 13;15:39805. doi: 10.1038/s41598-025-23469-y (PMC12615582; doi:10.1038/s41598-025-23469-y)
Supplement: Supplementary file 1 — Supplementary Material 1 [file 41598_2025_23469_MOESM1_ESM.docx]

**Supplementary Information File**

**Intraoperative hypotension after remimazolam or propofol induction with sevoflurane maintenance in angiotensin II receptor blockers-treated patients: a randomized controlled trial**

**Hye Jin Kim^1^, Namo Kim^1^, Jiho Kim****^1^, Jin Ha Park^1^, Hye Jung Shin^2^, Jinho Yang^1^, & So Yeon Kim^1,*^**

^1^ Department of Anesthesiology and Pain Medicine, Anesthesia and Pain Research Institute, Yonsei University College of Medicine, Seoul, Republic of Korea

^2^ Department of Biomedical Systems Informatics, Biostatistics Collaboration Unit, Yonsei University College of Medicine, Seoul, Republic of Korea

**Tables:**

**Supplementary Table S1** Classification of angiotensin II receptor blocker medications

| **Half-life** | **Propofol**  **(n=56)** | **Remimazolam**  **(n=56)** |
| --- | --- | --- |
| <12 h | 27 (48.2) | 32 (57.1) |
| Losartan | 8 (14.3) | 13 (23.2) |
| Valsartan | 9 (16.1) | 10 (17.9) |
| Fimasartan | 8 (14.3) | 6 (10.7) |
| Candesartan | 2 (3.6) | 3 (5.4) |
| 12–24 h | 9 (16.1) | 10 (17.9) |
| Irbesartan | 0 (0.0) | 3 (5.4) |
| Olmesartan | 9 (16.1) | 7 (12.5) |
| >24 h | 20 (35.7) | 14 (25.0) |
| Telmisartan | 20 (35.7) | 14 (25.0) |

Values are presented as the number of patients (percentage). *P*-value for the overall comparison between the propofol and remimazolam groups is 0.464

**Figure:**

**Supplementary figure S1** Mean arterial pressure decrease during anesthetic induction


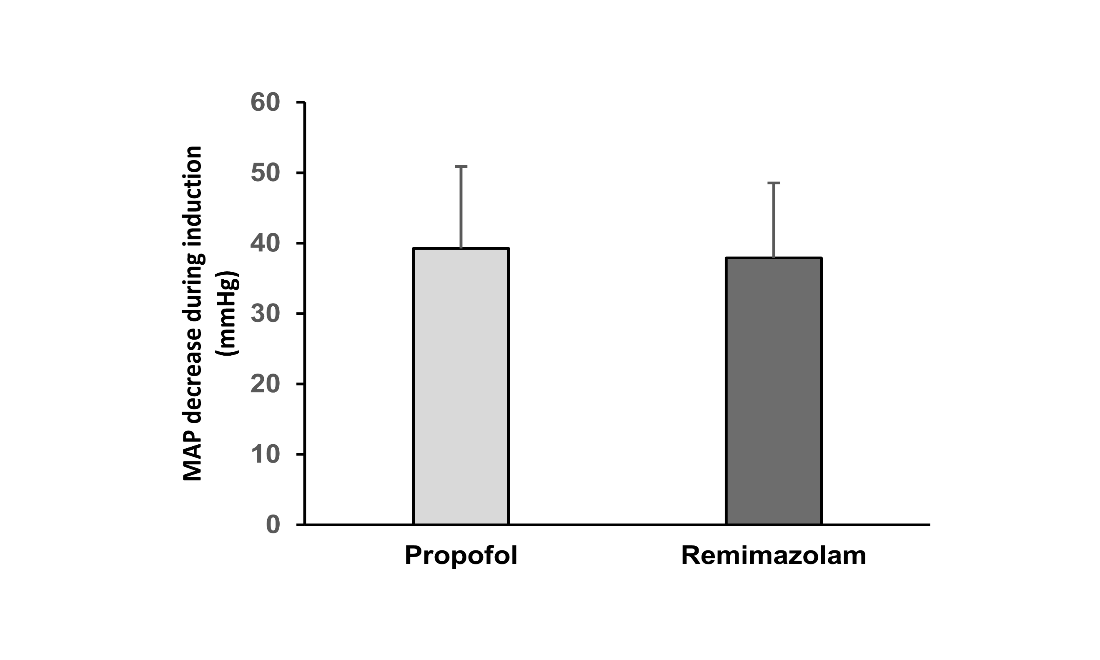


Mean arterial pressure (MAP) change was defined as the difference between MAP before induction and the lowest MAP within 15 minutes after induction. Bars represent mean ± SD. The mean MAP decrease was 39.2 ± 11.7 mmHg in the propofol group and 37.9 ± 10.7 mmHg in the remimazolam group, with no significant difference between groups (independent two-sample t-test, P = 0.527).
